# Microbial roles in cave biogeochemical cycling

**DOI:** 10.3389/fmicb.2022.950005

**Published:** 2022-09-28

**Authors:** Hai-Zhen Zhu, Cheng-Ying Jiang, Shuang-Jiang Liu

**Affiliations:** ^1^State Key Laboratory of Microbial Resources and Environmental Microbiology Research Center, Institute of Microbiology, Chinese Academy of Sciences, Beijing, China; ^2^College of Life Sciences, University of Chinese Academy of Sciences, Beijing, China; ^3^State Key Laboratory of Microbial Technology, Shandong University, Qingdao, China

**Keywords:** cave microbiome, biogeochemical cycling, cave evolution, mineral deposition, methane oxidation

## Abstract

Among fundamental research questions in subterranean biology, the role of subterranean microbiomes playing in key elements cycling is a top-priority one. Karst caves are widely distributed subsurface ecosystems, and cave microbes get more and more attention as they could drive cave evolution and biogeochemical cycling. Research have demonstrated the existence of diverse microbes and their participance in biogeochemical cycling of elements in cave environments. However, there are still gaps in how these microbes sustain in caves with limited nutrients and interact with cave environment. Cultivation of novel cave bacteria with certain functions is still a challenging assignment. This review summarized the role of microbes in cave evolution and mineral deposition, and intended to inspire further exploration of microbial performances on C/N/S biogeocycles.

## Introduction

Caves are dark, underground hollow spaces with relatively constant temperature, high humidity, and limited nutrients. Many caves are associated with karst topography, which is formed by the dissolution of soluble bedrock, such as limestone, dolomite and gypsum, in areas where groundwaters are undersaturated with respect to the minerals in the host rock. Karst landforms spread widely, accounting for approximately 20% of the earth’s dry ice-free surface (Ford and Williams, 2007). As a typical feature of subsurface landscape, karst caves develop globally, with over 50,000 distributed in the United States (Barton and Jurado, 2007). China also has a large contiguous karst terrain, and the Yunnan–Guizhou plateau in the southwest developed most karst caves, among which the longest cave exceeds 138 km (Zhang and Zhu, 2012). Many caves are relatively shallow and form near the water table in karst terranes, although some caves develop by deep-seated hypogenic process at substantial depths and by process other than dissolution such as lava flows.

**Figure 1:**
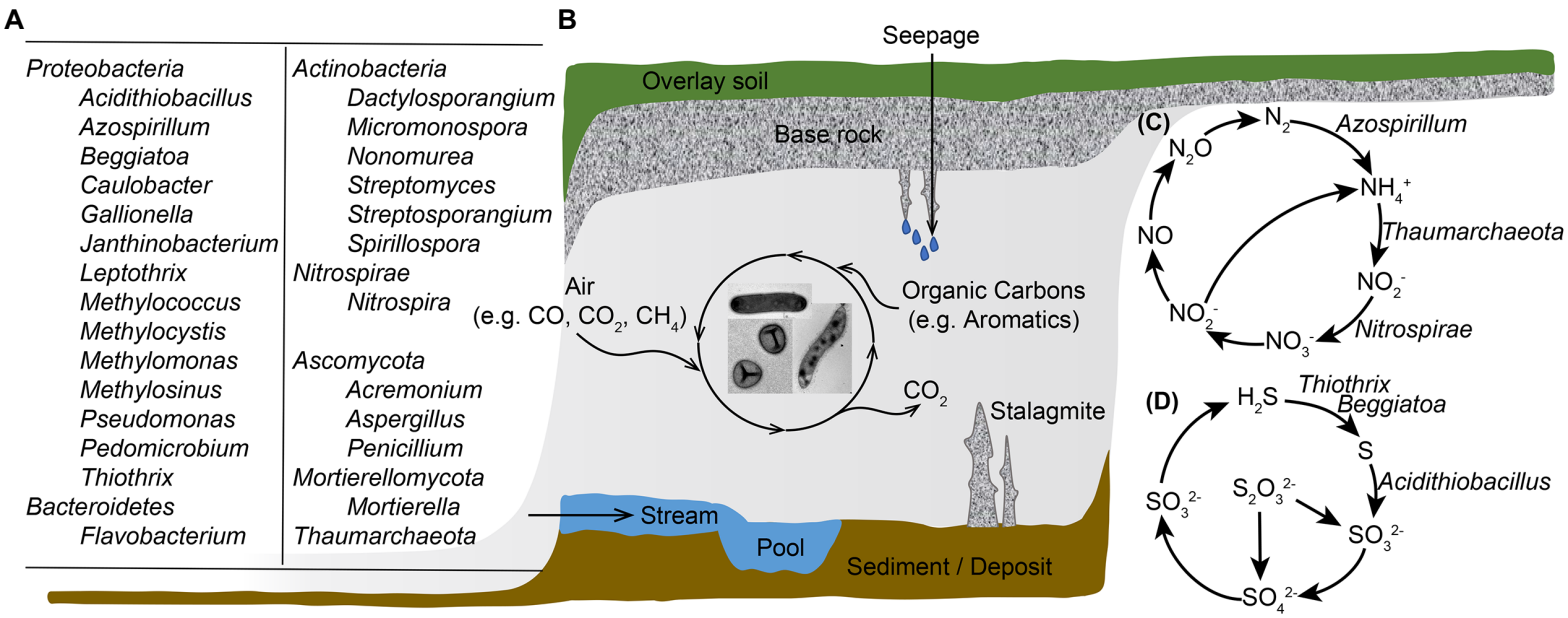
Illustration of microbial diversity and geobiochemical cycling of elements in a karst cave ecosystem. **(A)** Major cave microbial groups that are abundant and/or actively participate in biogeochemical cyclings; **(B)** Cave landscape and microbial carbon metabolisms; **(C)** Microbe-involved nitrogen transformation processes in caves; **(D)** Microbe-associated sulfur metabolisms in cave ecosystems. Microbes involved in goebiocycling processes in panels **(C,D)** are showed, except those genes were documented in cave samples yet no major microbial taxa responsible for the process was identified.

Caves are oligotrophic ecosystems with less than 2 mg of total organic carbon per liter, yet host flourishing microbial groups (Figure 1A), with an average number of 10^6^ microbial cells per gram of cave rock (Barton and Jurado, 2007). The study revealed a high diversity within *Bacteria* domain and *Proteobacteria* and *Actinobacteria* were abundant in oligotrophic cave samples of air, rock, sediment and water. *Chloroflexi*, *Planctomycetes*, *Bacteroidetes*, *Firmicutes*, *Acidobacteria*, *Nitrospirae*, *Gemmatimonadetes*, and *Verrucomicrobia* also accounted for large proportions of the total microbial community in caves (Wu et al., 2015; Zhu et al., 2019). In some organic cave samples such as biofilms in sulfur cave, bat guanos, spiders’ webs and earthworm castings, *Mycobacterium* was prevalently detected (Modra et al., 2017; Sarbu et al., 2018; Hubelova et al., 2021; Pavlik et al., 2021). Over 500 genera of fungi, such as *Penicillium*, *Aspergillus* and *Mortierella* have been reported in caves (Vanderwolf et al., 2013), and new fungal species were identified from cave air, rock, sediment and water samples (Zhang et al., 2017, 2021). These microbial communities contain novel diversity, and promote important biogeochemical processes. With no sunlight, microorganisms in cave environment cannot perform photosynthesis, and are intensively involved in the biogeochemical cycles of carbon, nitrogen, sulfur, and metals such as Fe and Mn to offset the lack of exogenous nutrients and energy.

Early studies of the participance of microbes in cave biogeochemical cyclings were carried out using traditional culturing techniques, and bacteria isolates participate in limestone calcification were obtained from cave deposits and pool waters (Danielli and Edington, 1983; Cunningham et al., 1995). Special attention was paid to cave *Actinobacteria*, as *Actinobacteria* members isolated from moonmilk deposits were proved to produce various novel antibiotic compounds (Axenovgibanov et al., 2016; Adam et al., 2018). The emerging new techniques greatly facilitated the investigations of cave microbial community structure and functional potential. By adopting metagenomic sequencing, chemolithotrophic microbial communities driven by nitrification and sulfur oxidation were identified from cave biofilms (Jones et al., 2012; Tetu et al., 2013). Based on both culture-dependent and culture-independent techniques, these studies expanded our knowledge of subsurface microbial diversity, provided a better understanding of the energy and nutrient dynamics of cave ecosystems.

Cave systems are geographically and geochemically complicated, and they might be formed from hydraulic (epigenic or hypogenic) or lava movements. Physical and chemical compositions of caves affect the microbial diversity and involvement in cave evolution. Readers who are interested in specificities of each cave system may refer to literatures (Palmer, 2011; Brannen-Donnelly and Engel, 2015; Martin-Pozas et al., 2020). In this review, we focus on discussion of the role of microbiomes in cave evolution and mineral deposition, and present case studies for microbial performance in cave nitrogen and sulfur cyclings. In addition, two emerging research topics related to cave carbon cycling, atmospheric methane oxidation and antibiotics production are explored.

## Microbiomes drive cave evolution

### Dissolution and deposition of carbonate minerals

There are two major mechanisms for the formation of karstic caves. Classical epigenic cave systems form as water flows through the soil and produces karst networks by seepages, absorbing CO_2_ and forming a dilute carbonate solution. In epigenic caves, exchanges occur with subaerial processes (such as water movement and mixture), enhancing dissolution capacity in the direction of flow (Sendra et al., 2014). In contrast, hypogenic cave systems form as water recharges the soluble bedrocks from below. The formation of hypogenic caves is fueled by hydrostatic pressure or other energy sources rather than recharge from the overlying or adjacent surface (Ford, 2006). Due to the deep origin of rising water, hypogenic cave systems are not directly influenced by seepages. Among explanations for hypogenic cave formation, sulfuric acid speleogenesis (the formation process of a cave) is a major one, resulting in some of the largest cave systems including Carlsbad Caverns and Lechuguilla Cave in New Mexico. Hydrogen sulfide leaked upward along with fractures from hydrocarbon deposits, and form sulfuric acid upon reaching oxygenated meteoric groundwater (Jagnow et al., 2000). Microbial communities dominated by sulfur-oxidizing acidophilic bacteria *Acidithiobacillus* formed dense biofilms in these caves, accelerating mineral dissolution and cave enlargement (Jones et al., 2014). Geobiochemical differences occur between epigenic and hypogenic cave systems, because caves formed from the two mechanisms would have different environmental conditions. For example, hypogenic caves such as sulfuric acid speleogenetic caves are sulfur-rich with relatively low pH, as a result, these caves support microbial communities that are acidophilic and actively involved in sulfur transformation. On the other hand, epigenic caves are pH neutral or slightly alkaline, and the microbial communities would be more diverse. Studies concerning snottites from sulfuric caves revealed very low biodiversity with Chao1 index ranged from 1 to 10 (Hose et al., 2000; Vlasceanu et al., 2000; Macalady et al., 2007), while more than ten thousand OTUs were detected from epigenic cave samples with Chao 1 index reached more than 1,000 (Zhu et al., 2019). Lava caves are also important subterrain environment, although karstic caves accounted for a larger proportion (Jones and Macalady, 2016). Lava caves formed from the heat volcanic flows and are mainly composed of basalt (Gabriel and Northup, 2013). These substantial differences from karstic caves granted lava caves unique microbial communities, which are mainly affected by geographical location and the availability of organic carbon, nitrogen and copper in the lava rock (Hathaway et al., 2014).

Multiple studies have suggested direct or indirect microbial involvement in the formation of carbonate minerals. Microbial calcite precipitation in calcium-rich environments was assumed to be the result of a detoxification process, through which growing cells actively export the excess calcium ions to maintain cellular metal homeostasis (Banks et al., 2010). Microbial biofilms also served as the initial crystal nucleation sites that contributed to the formation of secondary carbonate deposits within caves (Tisato et al., 2015). Cañaveras et al. (2006) proposed a model of moonmilk formation based on extensive observations in Altamira Cave (Spain) that microbial filaments provided a template for the precipitation of calcite fiber crystals in the early stages of moonmilk deposition. Moreover, the growth of microbes can increase the environmental pH, which would in turn increase the saturation index of carbonate and drive precipitation. For example, nitrogen metabolic pathways including ammonification of amino acids, dissimilatory reduction of nitrate and degradation of urea or uric acid induced formation of carbonate and bicarbonate ions, as the metabolic end product ammonia increased local pH (Castanier et al., 2000). Maciejewska et al. (2017) proved that *Streptomyces* promoted calcification in moonmilk through ammonification and, less importantly, ureolysis.

Biologically induced carbonate deposition was initially attributed mainly to fungi, studies reported that nano-fibers presented in the crystalline structure of moonmilk were related to biomineralized fungal hyphae (Bindschedler et al., 2010, 2014). However, recent studies indicated that bacteria played a major role in the induction of cave carbonate precipitation (Cañaveras et al., 2006; Portillo et al., 2009). γ-*Proteobacteria* and *Actinobacteria* were the major groups detected in white colonizations that were able to raise pH through metabolism in Altamira Cave (Portillo and Gonzalez, 2011); isolates belonging to α-*Proteobacteria*, β-*Proteobacteria*, γ-*Proteobacteria*, *Firmicutes* and *Actinobacteria* from cave speleothem were also confirmed to perform calcification (Banks et al., 2010). The presence of Archaea was identified in moonmilk deposits, yet their role in the formation of moonmilk remains to be discovered (Gonzalez et al., 2006; Reitschuler et al., 2016).

### Deposition of iron and manganese oxides

Fe and Mn oxides were found in karst caves as sedimentary fills, walls, ceiling and floor coatings/crusts, and sometimes as their own speleothems (Hill and Forti, 1997; Palmer, 2007). The importance of biological Fe oxidation in caves has long been recognized (Caumartin, 1963). Early studies on the bacterial role in Fe and Mn deposits most relied on microscopy: Peck first reported the presence of Fe-precipitating *Leptothrix* and *Gallionella* species in enrichment cultures inoculated with mud from cave pools and sumps (Peck, 1986). Later, more and more studies hinted the participance of microorganisms in cave Fe oxidation. For instance, freshly Fe oxide precipitates in Pautler Cave showed consistency with biomineralization structures of the microbial genera *Gallionella* and *Leptothrix* (Frierdich et al., 2011), and the iron mats in Borra caves appeared to be related to a community of mostly *Leptothrix*-like iron-oxidizing bacteria (Baskar et al., 2008, 2012). Abiotic Fe oxidation is rapid and prevalent at circumneutral pH yet is inhibited at low pH and microoxic/anoxic environments, where biological Fe oxidation more often occurs (Jones and Northup, 2021). The formation of Fe oxides and the identification of Fe-oxidizing bacteria do not necessarily indicate biological Fe oxidation.

Although Fe oxide minerals generate *via* both biological and abiotic processes, the presence of secondary Mn oxides is usually related to Mn-oxidizing microbes, because abiotic Mn oxidation is kinetically inhibited even at anoxic conditions (Luther, 2010; Johnson et al., 2013). Microbes could increase the rate of Mn oxidation by five orders of magnitude (Emerson, 2000; Tebo et al., 2005). Microbes related to Mn oxide production have long been reported from cave systems, and bacterial species belonging to *Proteobacteria*, *Firmicutes*, *Actinobacteria* and *Bacteroidetes* were reported to oxidize Mn (Carmichael and Bräuer, 2015). For example, Mn-oxidizing *Pseudomonas*, *Leptothrix*, *Flavobacterium* and *Janthinobacterium* species were isolated from cave ferromanganese deposits (Carmichael et al., 2013a); the well-known Mn-oxidizing *Pedomicrobium* and *Caulobacter* species were also observed in cave stromatolites (Lozano and Rossi, 2012). Most of the Mn oxidizers described to date are heterotrophic, they are supposed to oxidize Mn indirectly through producing superoxide during growth (Learman et al., 2011). However, Mn oxidation has long been regarded to be a potential energy-yielding reaction, and chemolithoautotrophic Mn oxidation was recently documented by Yu and Leadbetter (2020). A co-culture of two microbial species was obtained and the co-culture possibly coupled extracellular manganese oxidation to aerobic energy conservation and autotrophic carbon fixation (Yu and Leadbetter, 2020). Mn oxidation is largely influenced by exogenous carbon input, dilute sewage into the cave lead to massive bloom of a microbiome-driven and Mn-oxidizing biofilm (Carmichael et al., 2013b). Although bacteria attracted more attention in cave biogeochemical cycling, Mn-oxidizing fungi are also identified in caves. *Acremonium nepalense* was found responsible for black Mn oxides on clayey sediments of Lascaux Cave (Saiz-Jimenez et al., 2012); Mn-oxidizing members belonging to *Ascomycota* were obtained in southern Appalachian cave systems, and the results suggested that anthropogenic carbon sources stimulated fungi-driven Mn oxidation (Carmichael et al., 2015).

## The role of microbes in cave N cycle

Due to the dark and oligotrophic conditions, cave microorganisms use various metabolic pathways to retrieve nutrients and energy. In the world’s largest contiguous karst system, the Nullarbor Plain, extensive caves have been discovered (Webb and James, 2006). These caves are isolated from photosynthetically derived carbon, and no organic carbon was detected from filtered water flooding deeper portions of the caves (James and Rogers, 1994). However, dense biofilms with high biomass are widespread in Nullarbor caves, forming “mantles” of biological material associated with “snowfields” of microcrystals (Contos et al., 2001). Chemical analysis of water samples from Nullarbor caves showed relatively high levels of nitrite and remarkable sulfate and nitrate, indicating the existence of chemoautotrophic bacterial communities (Holmes et al., 2001). Holmes et al. (2001) also performed the first microbial community assessment of these slime biofilms, and revealed high proportion of clones belonging to *Nitrospira*, of which all characterized members carry the potential to oxidize nitrite into nitrate. These results suggested that nitrite oxidation could be essential to the trophic structure of Nullarbor cave communities. About 10 years later, Tetu et al. (2013) performed metagenomic and 16S rRNA amplicon sequencing of slime biofilms from one of the Nullarbor caves, providing in-deep knowledge of nitrogen transformations of these special communities. Their investigation indicated that *Thaumarchaeota* were abundant in the community, and *Thaumarchaeota* predominantly contributed to ammonia oxidation. Based on these studies, it was assumed that slime biofilms in Nullarbor caves had chemolithotrophic communities driven by nitrification. Except for caves with special biofilms like those in Nullarbor plain, sediment samples and ferromanganese deposits from other karstic caves also supported that *Thaumarchaeota* played an essential role in ammonia oxidation (Zhao et al., 2017; Kimble et al., 2018).

Studies with functional gene analysis of various cave samples documented other key N cycle pathways in addition to ammonia oxidation. For example, Kimble et al. (2018) found genes associated with nitrification, dissimilatory and assimilatory nitrate reduction, and denitrification in cave ferromanganese deposits with low fixed N; while Jones et al. detected nitrogen assimilation genes in cave biofilms where fixed N was available (Jones et al., 2012). In our previous work, a collection of cave bacterial genomes was established based on large-scale isolation and cultivation (Zhu et al., 2021). Except for many cave isolates carry genetic potential to perform denitrification and dissimilatory nitrate reduction, we also noticed that 11 genomes in the dataset showed the potential to fix nitrogen into biologically available ammonia. A novel nitrogen-fixing species *Azospirillum cavernae* was also identified from our cultured cave bacterial collections. These microbe-involved nitrogen transformation processes are summarized in Figure 1C.

## The role of microbes in cave S cycle

The biogeochemical cycle of sulfur instead of those of carbon and nitrogen was assumed being the center stage in sulfuric acid speleogenetic (SAS) caves (Hedrich and Schippers, 2021). Italy has about 25% of identified SAS cave systems worldwide, among which Frasassi cave system is the best documented and still active one (Galdenzi and Menichetti, 1995; D'Angeli et al., 2019). Extremely acidic (pH 0–1) microbial biofilms, which are known as “snottites,” hang on the walls and ceilings of these hydrogen sulfide-rich caves (Hose et al., 2000). Studies based on 16S rRNA cataloging showed that the Frasassi snottites were among the lowest diversities of natural microbial communities ever known, and were constituted mainly of bacteria related to *Acidithiobacillus* species, sometimes with other less abundant bacteria and archaea (Macalady et al., 2007). Jone et al. investigated the metabolic potential and ecological role of snottite *Acidithiobacillus* using metagenomics sequencing, and revealed that the population was autotrophic, and oxidizing sulfur *via* the sulfide-quinone reductase and *sox* pathways, indicating *Acidithiobacillus* was the snottite architect and main primary producer (Jones et al., 2012). Moreover, *Acidithiobacillus thiooxidans* obtained from the Frasassi cave snottites was also reported to carry a high potential to remove arsenic from contaminated sediments (Beolchini et al., 2017).

Microbial activity is also crucial for cave sulfide oxidation below the water table; the springs and discharge streams in SAS caves are colonized by thick, filamentous microbial mats (Engel et al., 2004b). Sulfide can be oxidized completely to sulfate with sufficient electron acceptors such as oxygen or nitrate; however, incomplete sulfide oxidization to sulfur would occur if low oxygen and nitrate are available. Due to intracellular or extracellular elemental sulfur globules formed by the partial oxidation of dissolved sulfide, biofilms in cave water have a milky appearance (Jones and Northup, 2021). Based on fluorescence *in situ* hybridization (FISH) and 16S rRNA gene libraries, ε-*Proteobacteria* and γ-*Proteobacteria* are crucial biofilm-forming members, and their distribution is primarily influenced by water flow (shear stress) and sulfide to oxygen ratios (Engel et al., 2003, 2004a). In rock-attached streamers, filamentous ε-*Proteobacteria* dominated at high while *Thiothrix* belonging to γ-*Proteobacteria* dominated at low sulfide to oxygen ratios; and in sediment–water interface, *Beggiatoa* belonging to γ-*Proteobacteria* was the dominant group regardless of sulfide to oxygen ratio (Macalady et al., 2008). Stream biofilms from Frasassi cave system were abundant in filamentous γ-*Proteobacteria*, among which *Beggiatoa*-like and/or *Thiothrix*-like cells contain large amount of sulfur inclusions (Macalady et al., 2006). Hamilton et al. (2015) retrieved *Sulfurovum*-like ε-*Proteobacteria* genomes through metagenomic sequencing of SAS cave biofilms, and indicated that this group is genetically equipped to catalyze sulfur precipitation while employing a lithoautotrophic lifestyle. Genes for the transformation of other sulfur-containing compounds were also reported in caves (Zhu et al., 2021), and these processes are summarized in Figure 1D.

## Featuring cave bioprocesses with C cycle

Due to limited nutrients, cave microbes have been reported to utilize diverse carbon and energy sources (Figure 1B). Our previous work noticed that genomes of cave bacterial isolates encode the genes for carbon monoxide oxidation (Zhu et al., 2021), a process that can either provide supplementary energy source without contributing biomass or couple with carbon dioxide fixation for biosynthesis under aerobic conditions (King and Weber, 2007). Carbon dioxide fixation is also active in cave microbial communities, there are six known carbon dioxide fixation pathways and the genes for all these pathways were detected in the metagenome of Kartchner cave surface (Ortiz et al., 2014). Aromatic hydrocarbons can be trapped by stalagmites or carried into caves through dripping water; consequently, bacteria that are able to degrade these compounds were detected in caves (Perrette et al., 2008; Marques et al., 2019). Microorganisms that are capable to use one-carbon compounds were obtained from Movile cave, and these microbes were proposed to be one of the main primary producers of the community (Wischer et al., 2015). In addition to traditional methanotrophs, the uncultured atmospheric methane-oxidizing bacteria were believed to be abundant in cave environment, which will be discussed detailly in the following part.

### Atmospheric methane oxidizers

As a potent greenhouse gas, the concentration of atmospheric methane is increasing (Nisbet et al., 2016; Prather and Holmes, 2017). Estimation showed that human activities and natural sources produce about 680 Tg year^−1^ of methane to the atmosphere, while reactions with hydroxyl and chlorine radicals in the troposphere and stratosphere remove about 600 Tg year^−1^ of methane (Kirschke et al., 2013). Methanotrophic microorganisms in forests, grasslands, paddy and other unsaturated soils play an essential role in mediating carbon cycle, and are believed to filter 30 Tg year^−1^ of methane (Kirschke et al., 2013). However, there is still gap in the overall methane budget balances. Recent studies suggested that caves and related karst terrains may be an essential yet overlooked sink for atmospheric methane.

Fernandez-Cortes et al. (2015) first monitored the concentration of methane and carbon dioxide in seven caves located in Spain, and the results proved that subterranean environments acted as sinks for atmospheric methane on seasonal and daily scales. They also detected methane-oxidizing bacteria in some cave sediments where methane concentrations were near to the atmospheric background, yet no such microbes were detected in sample sites with minimal methane concentrations. Thus, Fernandez-Cortes et al. assumed that cave methane oxidation was mainly induced by oxidative capacity from high density of ions, and was not significantly intervened by methanotrophic bacteria. However, through controlled laboratory experiment, Nguyễn-Thuỳ et al. (2017) showed that the radiolysis hypothesis is kinetically constrained and is unlikely to lead to rapid methane loss. Instead, by performing a set of mesocosm experiments with rock samples from two Vietnamese caves, they revealed that the depleted concentrations of methane in caves were most likely associated with microbial activity rather than radiolysis. Following these pioneering works, more and more evidence were reported from various caves to support microbes involved cave methane oxidation. For example, stable carbon and hydrogen isotope compositions of methane from 33 epigenic caves in the United States and 3 in New Zealand all supported that microbial methanotrophy within caves was the main methane consumption mechanism (Webster et al., 2018). Ojeda et al. (2019) also noticed methanotrophic activity of γ- and α-*Proteobacteria* in Nerja cave in Spain.

Methanotrophs have been reported since early twentieth century, and were detected at various habitats such as mud, rivers, rice paddies, sediments and sewage sludge (Whittenbury et al., 1970; Hanson and Hanson, 1996). Although atmospheric methane oxidation rates can remain steady for more than 4 months at 1.7 ppmv of methane (Schnell and King, 1995), calculations based on the kinetic constants of isolated methanotrophic bacteria could not support such extended survival (Conrad, 1984). Studies with *Methylosinus trichosporium* and *Methylobacter albus* revealed that atmospheric methane oxidation did not supply sufficient cellular maintenance energy and reduced power for the methane monooxygenase (Roslev and King, 1994; Schnell and King, 1995). The organisms responsible for atmospheric methane uptake were unknown until Holmes et al. (1999) reported a novel group of bacteria belonging to α-*Proteobacteria*, which is distantly related to existing methane-oxidizing strains and is believed to consume atmospheric methane. Later, Knief et al. (2003) identified another novel atmospheric methane-oxidizing group belonging to γ-*Proteobacteria* through comparative sequence analysis of the *pmoA* gene, and named these two groups as “upland soil cluster α” (USC α) and “upland soil cluster γ” (USC γ), respectively. Culture-independent studies suggested that USC α is adapted to the low concentration of methane in neutral and acidic soils (Kolb et al., 2005; Martineau et al., 2014) while USC γ prefers neutral to alkalic soils (Kolb, 2009). Tveit et al. (2019) reported the first pure culture *Methylocapsa gorgona* MG08 that grows on air at atmospheric methane, and proved that the strain is closely related to uncultured members of USC α. However, the cultivation of members related to USC γ remains challenging.

The investigation of cave methanotrophic bacteria is far earlier than the discovery that caves may be important methane sink. Hutchens et al. (2004) identified strains of *Methylomonas*, *Methylococcus* and *Methylocystis*/*Methylosinus* as major methanotrophs in Movile Cave through DNA-based stable isotope probing. However, the air of Movile Cave contains 1–2% (10,000–20,000 ppmv) methane (Sarbu and Kane, 1995), which is much higher than general 1.86 ppmv atmospheric level. Zhao et al. (2018) explored the presence and diversity of methane-oxidizing bacteria in Heshang Cave, where methane concentration decreases from 1.9 ppmv at the entrance to 0.65 ppmv inside the cave. Their results provided compelling evidence that methane-oxidizing bacteria accounted for up to 20% of the whole microbial communities with the high-affinity USC γ being the dominant group. According to sequencing analysis of *pmoA* and 16S rRNA genes of weathered rock samples from three karst cave in southwest China, Cheng et al. (2021) demonstrated that USC γ group dominated the atmospheric methane-oxidizing communities, and was identified as a keystone taxon in cooccurrence networks of both methane-oxidizing and the total bacterial communities. In addition to atmospheric methane-oxidizing groups, anaerobic methane-oxidizing bacteria were also detected from some cave samples. For example, members of the phylum *Rokubacteria* were found in Pindal Cave, these organisms perform anaerobic oxidation of methane coupled to nitrite reduction (Cuezva et al., 2020).

### Novel antibiotic compound producers from cave

Infectious diseases have long been threatening human society, while sprouting antibiotic-resistant pathogens are posing heavier burdens to human public health. According to the World Health Organization (WHO), there is an urgent need for investment of new antibiotics to combat antibiotic-resistant infections (World Health Organization, 2015). With a combination of unique environmental conditions and rare human intervention, more and more studies turned to caves for microorganisms producing novel antibiotics.

It has been proved that *Actinobacteria* are prolific producers of promising bioactive compounds with wide application. Approximately 45% of identified bioactive compounds are produced by *Actinobacteria*, among which 80% are derived from the *Streptomyces* genus (Bérdy, 2005). Sequencing analysis revealed the dominance of *Actinobacteria* in plenty of cave samples, such as cave soils (Wiseschart et al., 2019), cave sediments (De Mandal et al., 2015a,b), cave rocks (Zhu et al., 2019) and colonies on cave Paleolithic paintings (Stomeo et al., 2008). The isolates of *Streptomyces* genus were also obtained from many caves, such as Sigangli Cave in China (Fang et al., 2017), Altamira Cave and Tito Bustillo Cave in Spain (Groth et al., 1999), and Iron Curtain Cave in Canada (Gosse et al., 2019). Caves are not only home for various known actinobacterial taxa, but also excellent reservoir for new species of *Actinobacteria*. Forty-seven species within 30 genera belonging to *Actinobacteria* were isolated from caves and cave-related habitats from 1999 to 2018, among which seven represented novel genera (Rangseekaew and Pathom-aree, 2019). It is assumed that the extreme conditions within caves stimulated the inhabitant microorganisms to mutate their genes, making it more likely to evolve new species and novel metabolites (Tiwari and Gupta, 2013).

Isolating cave microorganisms and subsequently checking their antimicrobial activity against pathogens is one of the main approaches to identify novel antibiotics. By adopting such strategy, Herold et al. (2005) reported cervimycins, which are highly active against some Gram-positive bacteria. These compounds are produced by *Streptomyces tendae* HKI 0179, a strain isolated from a rock wall of an ancient cave in Italy. Another example is the discovery of Huanglingmycins, which are produced by *Streptomyces* sp. CB09001 from cave soil of China (Jiang et al., 2018). Noticeably, Huanglongmycin A showed not only weak activity against some Gram-negative bacteria, but also moderate cytotoxicity against A549 lung cancer cell line. Although certain bioactive compounds were not identified, some rare cave actinobacterium also showed anticancer potential. For instance, *Spirillospora albida* isolated from Phanangkhoi Cave were active against NCI-H1870 (human small lung cancer cell; Nakaew et al., 2009a); *Nonomurea roseola* isolated from Phatup Cave showed activity against human oral cavity cancer and human small lung cancer cells (Nakaew et al., 2009b). In addition, antifungal compounds were also documented from cave environment. Antagonistic *Streptomyces*, *Micromonospora*, *Streptosporangium*, and *Dactylosporangium* isolated from five caves in Korea showed biocontrol activities against at least one of the rice pathogenic fungi (Nimaichand et al., 2015); *Streptomyces* sp. from five caves in New Mexico and the United States has been suggested to inhibit the growth of the causative fungus of white-nose syndrome in bats (Hamm et al., 2017).

## Perspectives

Cave ecosystems form a huge subsurface reactor for the global biogeochemical cycle. The roles of microorganisms in both cave formation and subterranean key elements cycling are among the 50 top priority questions in subterranean biology (Mammola et al., 2020). It is now believed that many substantial mineral transformations, originally considered abiotic processes, are mediated by microbes: from microbial carbonate precipitation to the production of Fe and Mn deposits (Jones and Northup, 2021). As extremely starved environments, chemolithotrophic microbial communities driven by nitrification and sulfur oxidation have also been identified in cave ecosystems, providing valuable information on subterranean biogeochemical cycles (Jones et al., 2012; Tetu et al., 2013). These processes would not only transform minerals, change the air composition or water pH, but also lead to reshaping the cave. However, only 10% of all caves on earth have been accessed by humans, and even as many as 50% of caves in Europe and North America remain unexplored (Lee et al., 2012). As such, many more efforts are needed to explore cave microbiology, and further developments in caving technology and analytical tools are also essential to accomplish this goal.

The investigation about how microorganisms survive in nutrient-limited caves expanded our knowledge on controlling human impacts and protecting cave environments. For example, caves in north Spain contain Paleotic paintings, yet tourist activities brought in heterotrophic bacteria, threatening to damage the cultural treasures (Cañaveras et al., 2001). Furthermore, cave environment created stress for the inhabitant microorganisms at genetic level, making it a reservoir of novel microbial species and bioactive compounds. Although many cave bacterial isolates showed inhibitory properties against pathogens, only a few metabolites got identified chemical structure (Rangseekaew and Pathom-aree, 2019). The advent of genomics, transcriptomics and proteomics will facilitate the research of inter- and intra-community relationships which previously only be addressed under *in vitro* conditions. Nonetheless, to successfully isolate microorganisms that are adapted to cave environment and actively participate in element cycling is of vital importance yet remains challenging. Atmospheric methane oxidation in caves emerges as a hot research topic, although sequencing analysis proposed that USC γ played the major role, members of this group remain yet to be cultured (Cheng et al., 2021). On the one hand, a comprehensive understanding of factors affecting cave inhabitants is needed to discover ideal conditions for microbial growth; on the other hand, innovative cultivation techniques such as membrane diffusion-based cultivation, microfluidics-based cultivation and cell sorting-based cultivation are also worth applying (Lewis et al., 2020). By combining culture-dependent and sequencing-based techniques, cave microbiology explorations would lead to more exciting discoveries of subterranean environments.

## Author contributions

H-ZZ: writing the original draft. C-YJ and S-JL: discussion, editing, and finalizing the manuscript. All authors contributed to the article and approved the submitted version.

## Funding

This study was financially supported by the National Natural Science Foundation of China (grant no. 91951208).

## Conflict of interest

The authors declare that the research was conducted in the absence of any commercial or financial relationships that could be construed as a potential conflict of interest.

## Publisher’s note

All claims expressed in this article are solely those of the authors and do not necessarily represent those of their affiliated organizations, or those of the publisher, the editors and the reviewers. Any product that may be evaluated in this article, or claim that may be made by its manufacturer, is not guaranteed or endorsed by the publisher.
